# The roles of ING5 in cancer: A tumor suppressor

**DOI:** 10.3389/fcell.2022.1012179

**Published:** 2022-11-08

**Authors:** Hua-chuan Zheng, Hang Xue, Hua-mao Jiang

**Affiliations:** ^1^ Department of Oncology and Central Laboratory, The Affiliated Hospital of Chengde Medical University, Chengde, China; ^2^ Department of Urology, The First Affiliated Hospital of Jinzhou Medical University, Jinzhou, China

**Keywords:** signal pathway, biological function, ING5, cancer, tumor suppressor

## Abstract

As a Class II tumor suppressor, ING5 contains nuclear localization signal, plant homeodomain, novel conserved region, and leucine zipper-like domains. ING5 proteins form homodimer into a coil-coil structure, and heterodimers with ING4, histone H3K4me3, histone acetyltransferase (HAT) complex, Tip60, Cyclin A1/CDK2, INCA1 and EBNA3C for the transcription of target genes. The acetylated proteins up-regulated by ING5 are preferentially located in nucleus and act as transcription cofactors, chromatin and DNA binding functions, while those down-regulated by ING5 mostly in cytoplasm and contribute to metabolism. ING5 promotes the autoacetylation of HAT p300, p53, histone H3 and H4 for the transcription of downstream genes (Bax, GADD45, p21, p27 and so forth). Transcriptionally, YY1 and SRF up-regulate ING5 mRNA expression by the interaction of YY1-SRF-p53-ING5 complex with ING5 promoter. Translationally, ING5 is targeted by miR-196, miR-196a, miR-196b-5p, miR-193a-3p, miR-27-3p, miR-200b/200a/429, miR-1307, miR-193, miR-222, miR-331-3p, miR-181b, miR-543 and miR-196-b. ING5 suppresses proliferation, migration, invasion and tumor growth of various cancer cells *via* the suppression of EGFR/PI3K/Akt, IL-6/STAT3, Akt/NF-κB/NF-κB/MMP-9 or IL-6/CXCL12 pathway. ING5-mediated chemoresistance is closely linked to anti-apoptosis, overexpression of chemoresistant genes, the activation of PI3K/Akt/NF-κB and Wnt/β-catenin signal pathways. Histologically, ING5 abrogation in gastric stem-like and pdx1-positive cells causes gastric dysplasia and cancer, and conditional ING5 knockout in pdx1-positive and gastric chief cells increases MNU-induced gastric carcinogenesis. Intestinal ING5 deletion increases AOM/DSS- induced colorectal carcinogenesis and decreases high-fat-diet weight. The overexpression and nucleocytoplasmic translocation of ING5 are seen during carcinogenesis, and ING5 expression was inversely associated with aggressive behaviors and poor prognosis in a variety of cancers. These findings indicated that ING5 might be used for a molecular marker for carcinogenesis and following progression, and as a target for gene therapy if its chemoresistant function might be ameliorated.

## Introduction

Carcinogenesis is a complex biological process, and characterized by both frequent genetic and epigenetic changes, including the inactivation of tumor suppressor genes (TSG) and the activation of oncogenes. The chromosomal deletion, mutation and hypermethylation of TSGs result in their loss (Class I) and inactivation (Class II), finally to malignantly transform the normal cells. Inhibitor of growth (ING) family members belong to Class II TSG and play a critical role in initiation, promotion and development of cancers (Zheng et al., 2011; Dantas et al., 2019). In the review, we demonstrate that ING5 interacts with ING4, histone H3K4me3, HAT (histone acetyltransferase) complex, Tip60, Cyclin A1/CDK2, INCA1 and EBNA3C, and promotes the autoacetylation of HAT p300, p53, histone H3 and H4 for the transcription of downstream genes. Transcriptionally, YY1 and SRF up-regulate ING5 mRNA expression by the interaction of YY1-SRF-p53-ING5 complex with ING5 promoter. Translationally, ING5 is targeted by miR-196, miR-193a-3p, miR-27-3p and so forth. ING5 suppresses aggressive phenotypes of various cancer cells *via* EGFR/PI3K/Akt, IL-6/STAT3, Akt/NF-κB/NF-κB/MMP-9 or IL-6/CXCL12 pathway. Histologically, conditional ING5 abrogation causes gastric dysplasia and cancer, and increases chemically-induced gastrointestinal carcinogenesis. In human cancer samples, the up-regulated expression of ING5 was in gastric, breast, and colorectal cancers, but down-regulated in head and neck squamous cell carcinoma (HNSCC), lung cancer, osteosarcoma, prostate cancer, ovarian cancer, hepatocellular carcinoma (HCC), esophageal cancer, and thyroid cancer. These results suggest that ING5 might be used for a molecular marker for carcinogenesis and following progression, and as a target for gene therapy.

## The structure and functions of ING5

**FIGURE 1:**
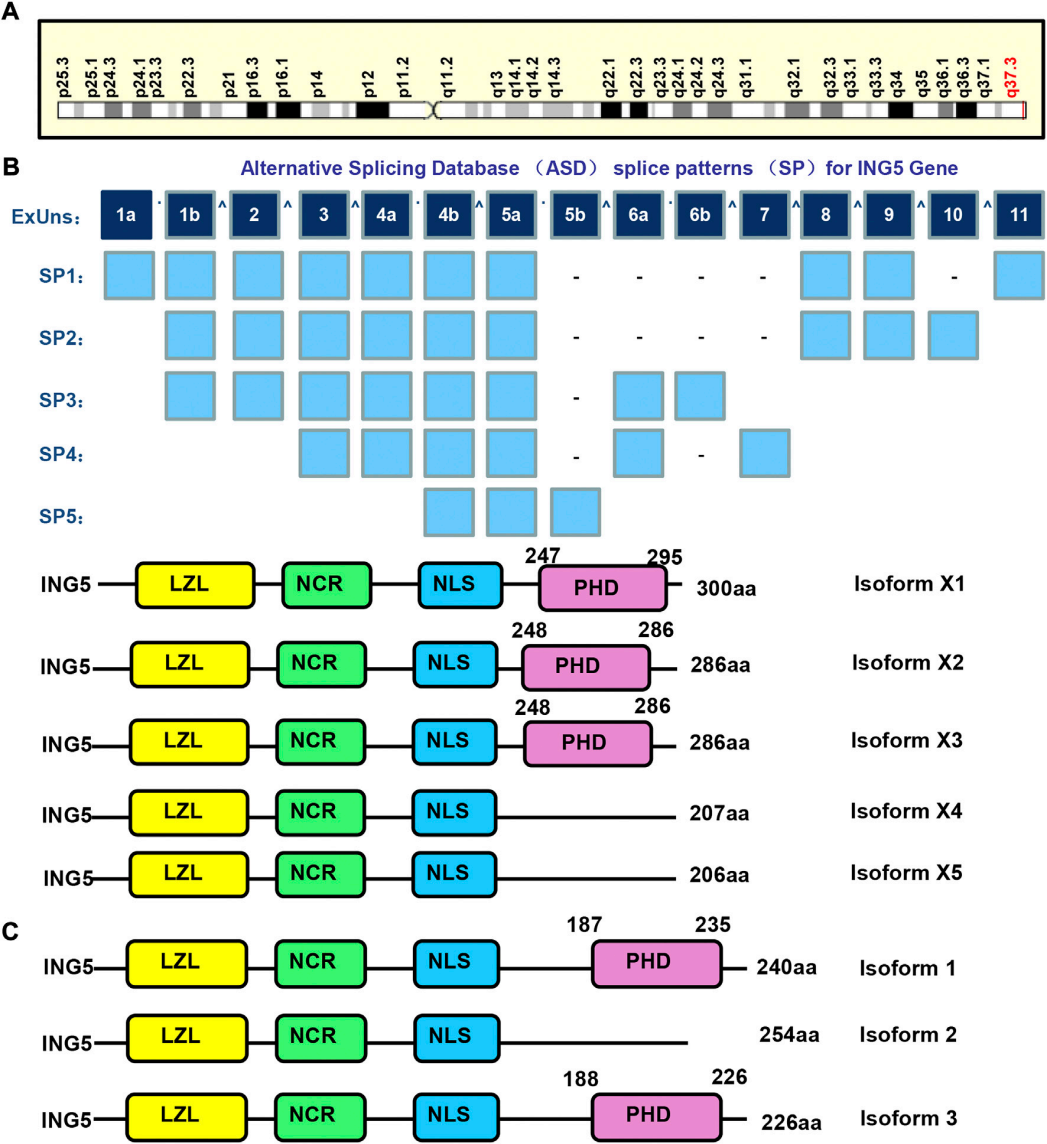
The structures of ING5 gene and protein. ING5 is localized in human chromosome 2q37.3 **(A)**, and theoretically spliced into 5 forms and encodes 5 proteins **(B)**. The 3 isoforms of ING5 are produced according to Genbank **(C)**. LZL, leucine zipper-Like motif; NCR: novel conserved region; NLS, nuclear localization signal; PHD, plant homeodomain.

The ING (inhibitor of growth) family is composed of ING1–5, and involved in apoptosis, senescence and DNA damage. They function as epigenetic readers of H3K4Me3 histone, and contribute to HAT or deacetylase complex formation. Their gatekeeper functions are evidenced by their arrest of the cell cycle, stabilization of p53, or maintenance of HAT activity. In contrast, their caretaker functions are evidenced by the nucleotide excision repair (NER) and double-strand break (DSB) repair of ING1-, and ING2- and ING3-mediated accumulation of ATM, BRCA1, and 53BP1 (Archambeau et al., 2019; Dantas et al., 2019). Among them, ING5 maps to human chromosome 2q37.3 (Figure 1A), contains 11 exons, theoretically spliced into 5 forms and encodes 5 proteins (Figure 1B). The 3 isoforms of ING5 are produced according to Genbank (Figure 1C). ING5 protein contains plant homeodomain (PHD), nuclear localization signal (NLS), novel conserved region (NCR), and leucine zipper-like (LZL) domains. Their NLS favors nucleic translocation, LZL mediates interaction with leucine zipper proteins, PHD interacts with such chromatin-interacting proteins as methylated histone H3, and NCR binds to HAT complex to remodel chromatin and modulate transcription (Zhang et al., 2017a; Archambeau et al., 2019; Dantas et al., 2019).

**FIGURE 2:**
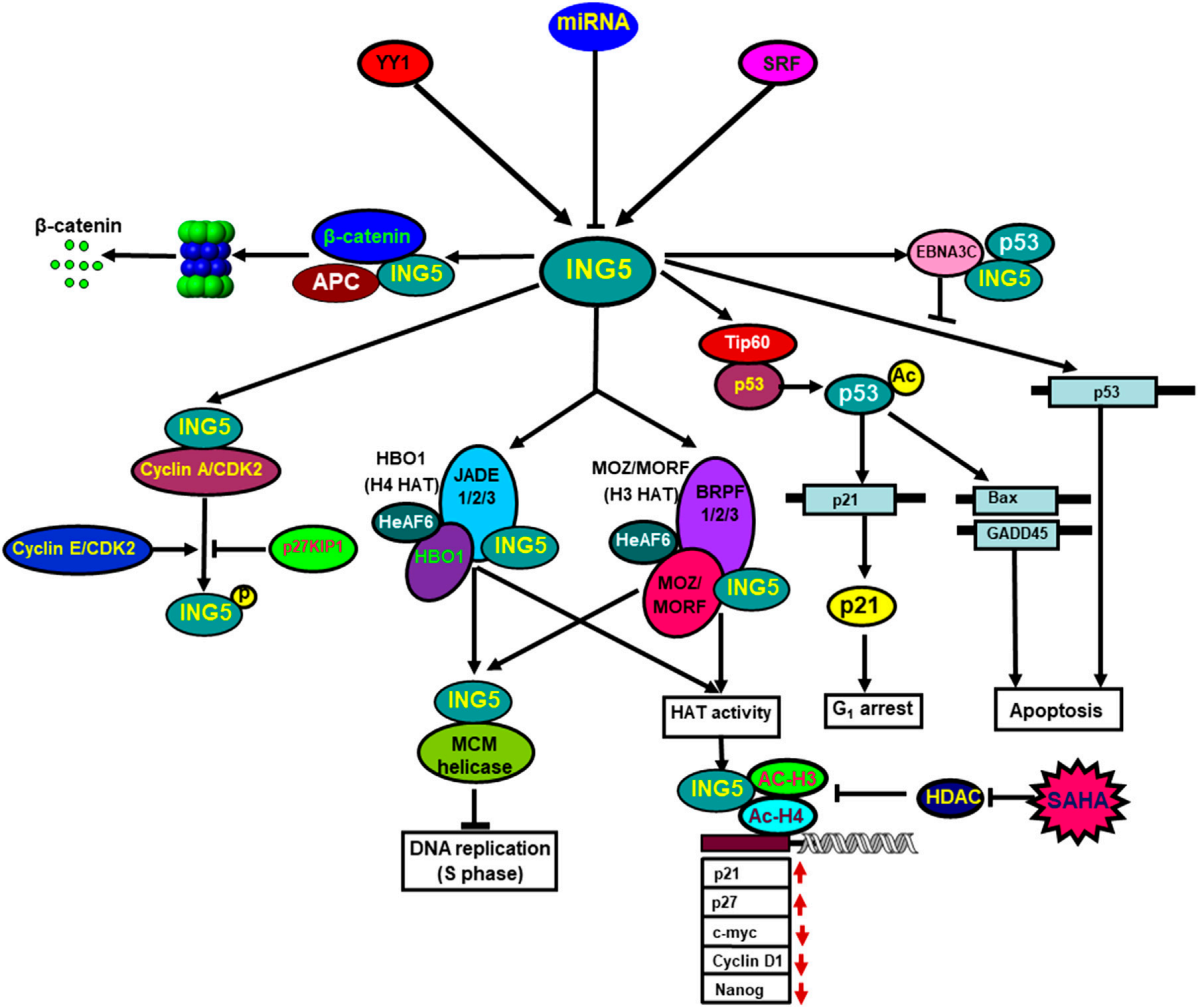
The biological functions of ING5. Transcriptionally, YY1 and SRF can up-regulate ING5 mRNA expression by the interaction of YY1-SRF-p53-ING5 complex with ING5 promoter. Translationally, ING5 expression is suppressed by as such miRNA targets as miR-196a, miR-193a-3p, miR-27-3p, miR-1307, miR-193, miR-222, miR-331-3p, miR-543 and so forth. ING5 interacts with histone H3K4me3 and is involved in the formation of two different HAT (histone acetyltransferase) complexes, (H4-HBO1-JADE-ING5 and H3-MOZ-MORF-BRPF-ING5), which contribute to DNA replication *via* ING5 and MCM complexes. ING5 binds to Cyclin A1/CDK2, and is phosphorylated by Cyclin E/CDK2 and Cyclin A/CDK2, which is repressed by CDK2 inhibitor p27KIP1. ING5 interacts with Epstain-Barr virus EBNA3C, which suppresses ING5-mediated transcription of p53. Additionally, ING5 assisted Tip60 to acetylate p53, and acetylated p53 at K120 binds to and activates the promoters of Bax and GADD45. ING5 also activates the p21 promoter to induce p21 expression *via* p53 acetylation at Lys-382. HAT and SAHA exposure recruited ING5 and acetylated histones H3 and H4 to the promoters of c-myc, nanog, Cyclin D1, p21, and p27, thereby regulating their transcription.

ING5 proteins form homodimer *via* their amino-terminal domain, and fold independently into a coil-coil structure. The PHD domains of the homodimer are chemically equivalent and independent of the rest of ING5. ING5 protein forms heterodimer with ING4 protein as well (Ormaza et al., 2019). ING5 binds to histone H3K4me3 and forms 2 HAT complexes, H3-MOZ-BRPF-MORF-ING5 and H4-JADE-HBO1-ING5. Both MCM and HAT complexes are involved in DNA replication because ING5 silencing abolishes DNA synthesis, and HBO1 silencing promotes the progression of S phase (Zhang et al., 2017a; Wu et al., 2018; Archambeau et al., 2019; Dantas et al., 2019; Ormaza et al., 2019). Zhang et al. (2011) identified ING5 as a partner of inhibitor of Cyclin A1 (INCA) that interacted with Cyclin A1/CDK2. Linzen et al. (2015) demonstrated that ING5 was phosphorylated at T152 by Cyclin E/CDK2 and Cyclin A/CDK2, which was repressed by CDK2 inhibitor p27KIP1. Wang et al. (2012) demonstrated ING5 suppressed the proliferation of mesenchymal stem cells by down-regulating CDK2 expression. ING5 and p53 bound to amino-terminal residues 129 to 200 of Epstein-Barr virus (EBV) EBNA3C. The conserved domain of ING5 competitively interacted with both p53 and EBNA3C. EBNA3C significantly inhibited ING5-mediated regulation of p53 transcription (Saha et al., 2011) (Figure 2).

ING5 profoundly influences protein lysine acetylation with 122 of 163 acetylation peptides significantly up-regulated, and 72 of 100 acetylation peptides down-regulated by ING5. The acetylated proteins up-regulated by ING5 are preferentially located in nucleus, and act as transcription cofactors, chromatin and DNA binding factors, while those down-regulated by ING5 are principally in cytosol, and involved in cellular metabolism. ING5 promotes HAT p300 autoacetylation at K1555, K1558, K1560, K1647 and K1794, resulting in the activation of HAT and subsequent acetylation of p53 at K382 and histone H3 at K18. HAT inhibitor impairs ING5-mediated acetylation of H3K18 and p53K382, and subsequently down-regulates both p21 and Bax expression (Zhang et al., 2017b). Ormaza et al. (2019) found that ING5 acetylated histone H3 by HBO1 complex, and H4 by MOZ/MORF complex. Liu et al. (2013) found that ING5 facilitated Tip60 to acetylate p53 at K120 in response to DNA damage by the formation of p53-Tip60 complex. Acetylated p53 at K120 interacted with and activated the promoters of the apoptotic genes, Bax and GADD45. ING5 was reported to activate the cyclin-dependent kinase inhibitor p21 promoter and up-regulate p21 expression, and acetylate p53 protein at K382 (Shiseki et al., 2003) (Figure 2). Wang et al. (2018) found that ING5 enhanced the expression of stem cell markers (Oct4, Olig2 and Nestin) to promote self-renewal, prevent lineage differentiation and increase stem-like population of brain tumor initiating cells.

## The biological effects of ING5 expression on cancer cells

ING5 inhibits proliferation, glucose catabolism, anti-apoptosis, migration, invasion, epithelial- mesenchymal transition (EMT) or lung metastasis, and induces cell arrest, senescence, autophagy, fat accumulation, and chemoresistance in gastric, colorectal, breast, lung, ovarian cancer cells, neuroblastoma and glioma cells (Gou et al., 2015; Zhao et al., 2016; Ding et al., 2017; Zhao et al., 2017; Zheng et al., 2017; Wu et al., 2018; Yu et al., 2022; Zheng et al., 2022). ING5 inhibited tumor growth, blood supply or lung metastasis of gastric, colorectal, ovarian, breast or lung cancer cells by suppressing proliferation, and inducing autophagy and apoptosis in nude xenograft models. ING5-mediated chemoresistance was closely linked to anti-apoptosis, overexpression of chemoresistant genes, the activation of PI3K/Akt/NF-κB and Wnt/β-catenin signal pathways (Gou et al., 2015; Zhao et al., 2016; Ding et al., 2017; Zhao et al., 2017; Zheng et al., 2017; Wu et al., 2018; Yu et al., 2022). Qi and Zhang (2014) found that intact ING5 inhibited the proliferation and anti-apoptosis in tongue squamous cell carcinoma cells. In addition, 2 mutants of ING5 (aa 107-226 and 1-184) can facilitate cellular senescence with Cyclin E and CDK2 hypoexpression. In our study, we found that all wild-type and mutant ING5 had either the LZL or PHD domain, and weakened proliferation, migration and invasion of gastric cancer cells, which was closely linked to cdc-2, VEGF, and MMP-9 hypoexpression (Zheng et al., 2022).

ING5 was also demonstrated to inhibit proliferation, glucose catabolism, migration, invasion, EMT, and tumor growth, and promote apoptosis, cell cycle arrest, senescence, or autophagy in prostate cancer cells by activating p53 and inactivating Akt (Barlak et al., 2020), in lung cancer cells by phosphorylating β-catenin at S33/37, and suppressing IL-6/STAT3 and EGFR/PI3K/Akt pathways (Zhang et al., 2015; Liu et al., 2017; Liu et al., 2019), in neuroblastoma cells by acetylating histones (Wu et al., 2018), in colorectal cancer cells through PI3K/Akt pathway (Xin et al., 2020), in osteosarcoma cells *via* Smad pathway (Zhang et al., 2018a), in esophageal squamous carcinoma cells through IL-6/CXCL12 (Wang et al., 2021) and Akt/NF-κB/MMP-9 (Zhang et al., 2018b) pathways, and in breast cancer cells *via* PI3K/Akt/NF-κB pathway (Zhao et al., 2015; Cui et al., 2017), in glioma cells (Zhao et al., 2017) and gastric cancer cells (Gou et al., 2015) *via* either β-catenin/TCF-4 or PI3K/Akt pathway, and thyroid cancer *via* c-Met/PI3K/Akt signaling pathway (Gao and Han, 2018). These findings suggest that ING5 reverses the aggressiveness mostly through PI3K/Akt signal pathway.

Wang et al. (2018) reported that ING5 promoted stemness and self-renewal, and prevented lineage differentiation in glioblastoma cells *via* Ca^2+^ and follicle-stimulating hormone. Chen et al. (2017) demonstrated that ING5 inhibited cell proliferation and chemoresistance, and promoted cell apoptosis in ovarian cancer cells. Li et al. (2015) found that ING5 silencing increased the chemoresistance and inhibited the DNA damage response pathway in bladder cancer, while it was the converse for ING5 expression in chemoresistant bladder cancer cells. Our previous work showed that ING5 overexpression caused chemoresistance and lipogenesis of cancer cells, and chemoresistant cells to 5-FU, cisplatin and sorafenib showed ING5 overexpression and lipogenesis, suggesting that ING5-mediated lipogenesis results in drug resistance (Xuan et al., 2022).

ING5 knockdown elevated cell viability and impaired cell cycle G_0_/G_1_ phase arrest and apoptosis in PC-12 and SH-SY5Y cells after oxygen-glucose deprivation (Zhang et al., 2019). Wu et al. (2018) demonstrated that HAT recruited ING5 and acetylated histones H3 and H4 to the promoters of c-myc, nanog, Cyclin D1, p21, and p27, thereby up-regulating p21 and p27 expression, and down-regulating c-myc, Cyclin D1 and nanog expression. SAHA treatment attenuated the histone deacetylase inhibitor and finally strengthened the formation of ING5-acetyl- H3-acetyl-H4 (Figure 2).

## The regulation of ING5 expression

At the transcriptional level, our group found out a suppressor between −2000 and −1000 bp, and an enhancer between −800 and −100 bp although two promoter sequences were predicted from -1995 to −1690 bp and from -1973 to −1400 bp. The trans-acting factors of ING5 promoters are predicted to be SRF (−717 to −678 bp), CTCF (−48 to 0 bp), YY1 (−48 to −25 bp), Sp1 (−44 to −30 bp; 32 to 20), PPAR-γ1 (−24 to −25 bp), WT1 (−10 to 1 bp), and Pax-5 (−1 to 25 bp). EMSA and ChiP indicated that only SRF and YY1 could interact with the *ING5* promoter (−50 to 0 bp). In gastric cancer cells, either SRF or YY1 silencing decreased *ING5* mRNA and protein expression. SRF was found to interact with YY1, p53 and ING5, and either SFR or YY1 was co-localized with p53 or ING5 in the nuclei of cancer cells (Zheng et al., 2022). These findings indicate that SRF, YY1, ING5 and p53 form a complex to interact with the ING5 promoter and up-regulate its expression in gastric cancer cells.

At the translational level, both miR-196 and miR-196b-5p target ING5 in colorectal cancer cells (Pourdavoud et al., 2020; Xin et al., 2020). By targeting ING5, miR-27-3p promotes the G_1_-S phase transition in osteosarcoma cells (Ye et al., 2018) and miR-196b-5p inhibits the apoptosis of pancreatic cancer cells (Ma et al., 2021). In ovarian cancer cells, miR-200b/200a/429 targets ING5 which blocks miR-200b/200a/429-induced malignant transformation (Guan et al., 2020), and miR-1307 targets and down-regulated ING5 expression (Chen et al., 2017). miR-1307- mediated apoptosis and chemosensitivity were *in vivo* and vitro reversed by ING5 knockdown. CAF (cancer-associated fibroblast)-derived exosomal miR-196a confers cisplatin resistance in HNSCC cells by targeting ING5 (Qin et al., 2019), and miR-193a-3p induces the chemoresistance of breast cancer cells by targeting ING5, in line with the effects of ING5 silencing (Li et al., 2015). In triple-negative breast cancer cells, miR-193 bound to the 3′-UTR of ING5 mRNA to inhibit its expression and miR-193 inhibitor suppressed cell proliferation through ING5/PI3K/Akt pathway (Xu et al., 2020). CASC2 acted as a competitor of miR-222 to up-regulate ING5 expression in pulmonary arterial smooth muscle cells (PASMCs). CASC2- mediated inhibitory effects of hypoxia on the migration and proliferation of PASMCs could be weakened by either miR-222 inhibitor or ING5 overexpression (Han et al., 2020). miR-331-3p could promote proliferation of HCC cells by targeting ING5, which was strengthened by hepatitis B virus (HBV) (Cao et al., 2015). HBx protein of HBV accelerated the proliferation of HCC cells by up-regulating miR-181b to target ING5 (Xie et al., 2018). In neuroblastoma cells, ING5 is a negatively- regulated target gene of miR-376c-3p (Zhang et al., 2019), and SAHA down-regulates miR-196-b and miR-543 expression to facilitate the translation of ING5 protein (Wu et al., 2018). miR-193 induces proliferation and CDK2 activity in bone mesenchymal stem cells by targeting ING5 (Wang et al., 2012).

## The effects of ING5 knockout on carcinogenesis and lipogenesis

ING5 was conditionally deleted in gastric pit cells using Capn8-cre mice, in gastric parietal cells using Atp4b-cre mice, in gastric pdx-1-positive cells using pdx1-cre mice, in gastric stem-like cells using K19-cre mice, in gastric chief cells using PGC-cre mice, and in intestinal epithelial cells using villin-cre mice. The normal gastric epithelium was observed in Capn8-cre; ING5^f/f^ mice and regenerative dysplasia was detected in PGC-cre; ING5^f/f^ mice. However, well-, moderately-, or poorly-differentiated adenocarcinoma was observed in Atp4b-cre; ING5^f/f^ ,pdx1-cre; ING5^f/f^ and K19-cre; ING5^f/f^ mice, even in pdx1-cre; ING5^f/f^ and K19-cre; ING5^f/f^ and wild-type (WT) mice exposed to MNU. After the treatment with MNU, chemically-induced gastric cancer and dysplasia were more common in K19-cre; ING5^f/f^ and Pdx1-cre; ING5^f/f^ mice than those in WT mice. These data demonstrated that ING5 knockout (KO) might cause spontaneous and chemically-induced gastric carcinogenesis (Zheng et al., 2022). After exposure to AOM/DSS, the cancer formation rate of intestinal KO mice was high in comparison to that of WT mice (Yu et al., 2022).

After the oral administration of high-fat diet, villin-cre; ING5^f/f^ showed a lower weight, a slighter fatty liver and a lighter fat weight than WT mice (Yu et al., 2022). He et al. (2021) found that liver-specific reconstitution of JFK expression resulted in hepatic lipid accumulation in JFK KO mice by destabilizing ING5 and inhibiting fatty acid β-oxidation. Therefore, we hypothesized that ING5 might play an important role in fat absorption and fatty acid β-oxidation.

## The clinicopathological and prognostic significances of ING5 in cancers

**FIGURE 3:**
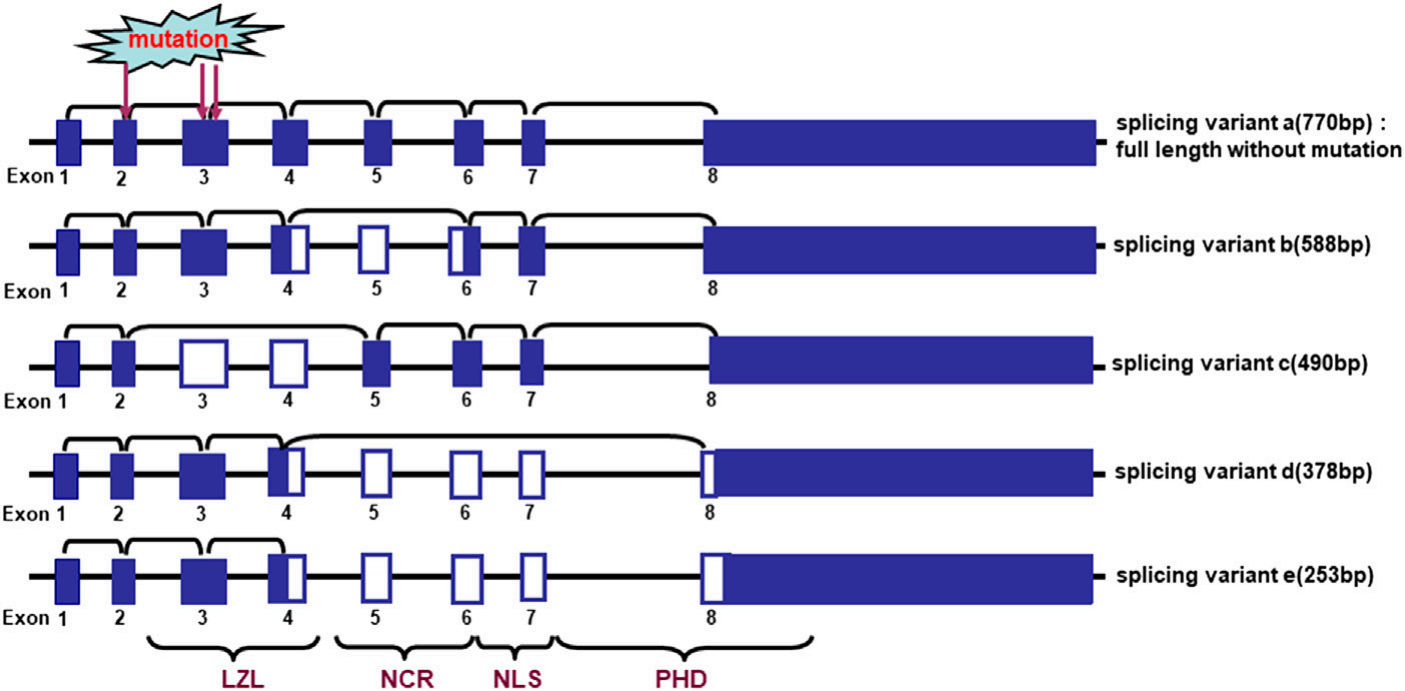
Alternative splicing of ING5 mRNA in oral squamous cell carcinoma. The schematic representation indicates the genomic structure and alternatively splicing variants of ING5. LZL, leucine zipper-Like motif; NCR: novel conserved region; NLS, nuclear localization signal; PHD, plant homeodomain.

Cengiz et al. (2007) found that 3 frequently-deleted regions at 2q37.3, 2q33-35 and 2q21-24 harboring ING5 were detected in oral cancers, and loss of heterozygosity (LOH) of chromosome 2q in 85% (33/39) of oral squamous carcinomas, indicating an important role of ING5 deletion in oral carcinogenesis (Cengiz et al., 2010). There were 5 different alternative splicing variants of ING5, which resulted in ING5 mRNA hypoexpression in 61% of the oral tumors as compared to the matched normal samples (Figure 3). These missense mutations of ING5 gene were located to NCR and LZL domains of ING5 protein (Cengiz et al., 2007; Cengiz et al., 2010).

In our previous study, ING5 protein is primarily observed the cytoplasm and nucleus in the breast, intestine, stomach, and tongue, lung. Totally, ING5 expression was detected in 40.6% (400/986) of cancer tissues, among which ING5 was more frequently expressed in colorectal (56.3%), breast (79.9%) and endometrial (50.0%) cancers, and less in pancreatic cancer (22.6%) and HCC (14.5%) (Yang et al., 2019). Although a high ING5 expression was observed in gastric, breast, and colorectal cancers (Xing et al., 2011; Zhao et al., 2015; Wang et al., 2021; Zheng et al., 2022), ING5 was found to be expressed in osteosarcoma, prostate cancer, ovarian cancer, HCC, lung cancer, esophageal cancer, and thyroid cancer at a low level (Qi and Zhang, 2014; Cao et al., 2015; Zhang et al., 2015; Cui et al., 2017; Zheng et al., 2017; Zhang et al., 2018a). The low nuclear expression of ING5 and its nucleocytoplasmic translocation were involved in the tumorigenesis of gastric cancer, breast cancer, HNSCC, and colorectal cancer (Li et al., 2010; Xing et al., 2011; Zheng et al., 2011; Ding et al., 2017). Bilgiç et al. (2018) found that ING5 was under-expressed in the atrophic gastritis and gastric cancer compared with the normal mucosa.

As for the clinicopathological and prognostic significances, ING5 mRNA expression was negatively correlated with vascular and lymphatic invasion, and clinicopathological staging of ovarian cancers. There was an adverse relationship between ING5 mRNA expression and the overall or progression-free survival of the ovarian cancer patients with stage I, Grade 3, and Grade 2, indicating the prognostic significance of ING5 depends on the cancer type, subtyping and molecular level (Zheng et al., 2017). In gastric, colorectal and breast cancers, nuclear ING5 expression was negatively associated with depth of invasion, tumor size, dedifferentiation, and TNM staging, opposite to the finding of cytoplasmic ING5 expression (Xing et al., 2011; Zheng et al., 2011; Ding et al., 2017). ING5 immunoreactivity was less expressed in ovarian mucinous and serous adenocarcinoma than miscellaneous subtypes, and negatively correlated with differentiation and low ki-67 expression of ovarian cancer (Zhang et al., 2018b). In lung cancer, ING5 protein was distinctly expressed in small cell carcinoma < large cell carcinoma < adenocarcinoma < squamous cell carcinoma (Zhao et al., 2016). The nuclear ING5 expression was positively linked to a better prognosis of gastric cancer patients, albeit not independent (Xing et al., 2011), in agreement with the report of Zhang et al. about lung cancer (Zhang et al., 2015).

## Conclusion and perspectives

In summary, ING5 protein can acetylate HAT p300, p53, histone H3 and H4 for the transcription of downstream genes *via* the formation of homodimer or heterodimers with ING4, histone H3K4me3, HAT complex, Tip60, Cyclin A1/CDK2, INCA1 and EBNA3C. Transcriptionally, YY1 and SRF can up-regulate ING5 mRNA expression by the interaction of YY1-SRF-p53-ING5 complex with ING5 promoter. ING5 inhibits proliferation, migration, invasion and tumor growth of cancer cells *via* IL-6/STAT3, EGFR/Akt/NF-κB/MMP-9 or IL-6/CXCL12 pathways. ING5-mediated lipogenesis might lead to chemoresistance. Target ING5 KO increased the sensitivity of chemically-induced gastric and colorectal carcinogenesis, and suppressed lipogenesis and fat absorption in intestine. The overexpression and nucleocytoplasmic translocation were seen and ING5 expression is inversely associated with aggressive behaviors and poor prognosis of cancers.

In the future, aberrant ING5 expression might be used as a molecular marker for carcinogenesis, progression and prognosis of malignancies. Additionally, ING5 functions as a tumor suppressor, but facilitates lipogenesis and subsequent chemoresistance. If ING5 will be used as a gene therapy target of cancers, the chemoresistant function should be ameliorated. Therefore, ING5 knockout or the suppression of ING5-related signal pathway would also be helpful to the control of body weight and the prevention of obesity-related diseases. If we can develop the anti-ING5 reagents, these drugs would be used to treat cancer, reverse chemoresistance and control the obesity.

## Abbreviations

CAF, cancer-associated fibroblast; DSB, double-strand break; EBV, Epstain-Barr virus; EMT, epithelial- mesenchymal transition; HAT, histone acetyltransferase; HBV, hepatitis B virus; HCC, hepatocellular carcinoma; HNSCC, head and neck squamous cell carcinoma; ING, inhibitor of growth; KO, knockout; LOH, loss of heterozygosity; LZL, leucine zipper-like; NER, nucleotide excision repair; NCR, novel conserved region; NLS, nuclear localization signal; PASMC, pulmonary arterial smooth muscle cell; PHD, plant homeodomain; WT, wild-type.
